# 12 year follow up of enzyme-replacement therapy in two siblings with attenuated mucopolysaccharidosis I: the important role of early treatment

**DOI:** 10.1186/s12881-016-0284-4

**Published:** 2016-03-10

**Authors:** Orazio Gabrielli, Lorne A. Clarke, Anna Ficcadenti, Lucia Santoro, Lucia Zampini, Nicola Volpi, Giovanni V. Coppa

**Affiliations:** Pediatric Division, Department of Clinical Sciences, Polytechnic University of Marche, Ospedali Riuniti, Presidio Salesi, Via Corridoni 11, 60123 Ancona, Italy; Child and Family Research Institute, Department of Medical Genetics, University of British Columbia, Vancouver, Canada; Department of Biology, University of Modena & Reggio Emilia, Modena, Italy

**Keywords:** Attenuated Mucopolysaccharidosis type I, Enzyme replacement therapy, α-L-iduronidase, laronidase

## Abstract

**Background:**

Mucopolysaccharidosis type I is an autosomal recessive disorder caused by deficiency of α-L-iduronidase and characterized by a progressive course with multisystem involvement. Clinically, Mucopolysaccharidosis type I is classified into two forms: severe (Hurler syndrome), which presents in infancy and is characterized by rapid progressive neurological involvement and attenuated (Hurler/Scheie and Scheie syndromes), which presents with slower progression and absent to mild nervous system involvement. The specific treatment for attenuated Mucopolysaccharidosis type I consists of enzyme-replacement therapy with laronidase (human recombinant α-L-iduronidase, Aldurazyme). We present here the clinical and laboratory results in an 12-year-old patient affected by the attenuated form of Mucopolysaccharidosis type I treated by enzyme-replacement therapy from the age of 5 months, compared with his 17 year old affected sister, who started therapy at 5 years of age.

**Case Presentation:**

Clinical evaluation of these siblings shows that initiation of therapy prior of the onset of clinically detectable disease resulted in considerable improvement in outcome in the young sibling. After 12 years of enzyme-replacement therapy, facial appearance, linear growth rate, and liver and spleen volumes were normal; moreover, the degree of joint disease, vertebral, and cardiac valvular involvement were only minimal compared with those of his sister.

**Conclusion:**

This study demonstrates that early diagnosis and early initiation of enzyme-replacement therapy substantially modify the natural history of the attenuated form of Mucopolysaccharidosis type I.

## Background

Mucopolysaccharidosis type I (MPS I) is an autosomal recessive disorder caused by the deficiency of α-L-iduronidase and characterized by a progressive multisystem disease with ophthalmologic, cardiac, gastrointestinal, pulmonary, skeletal and nervous system involvement. Although there is considerable clinical heterogeneity, patients fit into two broad clinical phenotypes: severe, also known as Hurler syndrome, and attenuated, previously termed Hurler/Scheie and Scheie syndromes [1]. Hurler syndrome presents in early infancy and is characterized by early and progressive multisystem disease including neurological involvement and, when untreated, leads to death within the first decade of life. Attenuated MPS I patients show considerable clinical heterogeneity, with symptom presentation most commonly in the mid to late childhood, but definitive diagnosis can be delayed into the second decade. Patients with attenuated disease show slower but relentless multisystem disease progression with absent to mild primary central nervous system involvement. Life expectancy for untreated attenuated MPS I patients ranges from the second decade to normal life expectancy.

Enzyme replacement therapy (ERT) with laronidase, recombinant human α-L-iduronidase (Aldurazyme) has become the standard of care for attenuated MPS I patients [2]. We present here a 12 year follow up of a previously reported patient with attenuated MPS I [3] who started ERT at the age of 5 months, in comparison with his older sister who started the therapy at 5 years of age. To our knowledge, there are no other patients treated before 6 months of life and followed for this extended period of 12 years.

## Case presentation

### Siblings

This brother and sister pair are currently 12 years, and 17 years of age, respectively. The sister was diagnosed with MPS I at the age of 5 at which time she had classic features of attenuated MPS I. The diagnosis in the sister led to the diagnosis in the brother shortly after birth. Full case details can be found in the previous publication [3]. Both siblings are compound heterozygotes for the *IDUA* mutations W402X and L535F. Complete clinical and biochemical evaluations have been performed annually on both children. Each commenced weekly laronidase infusions at a dose of 0.5 mg/kg at the ages of 5 months (brother) and 5 years (sister). Infusions took place in hospital with the use of implanted venous access devices; they were well tolerated with no reported reactions and in addition very few infusions were missed.

### Sibling 1

**Fig. 1:**
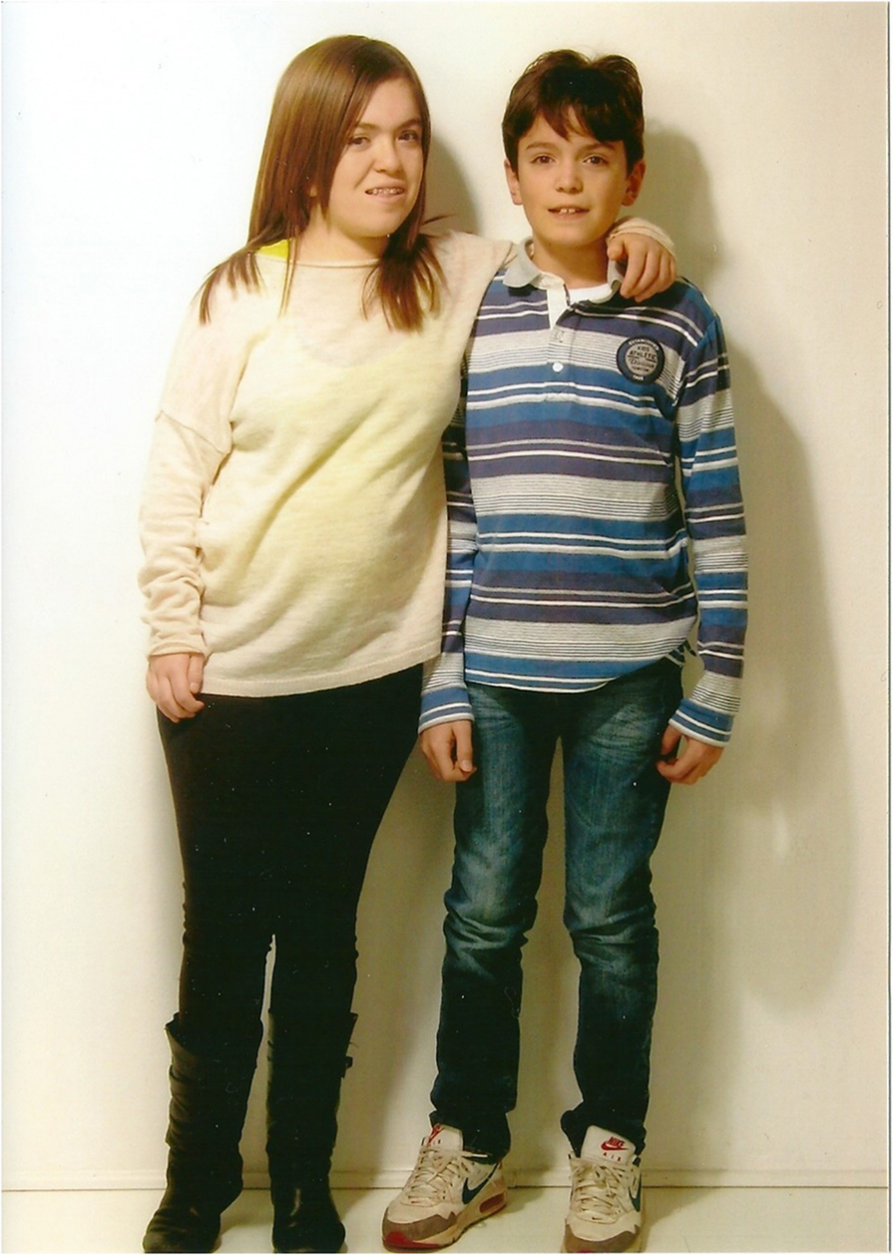
Patient M at the age of 10 years and Patient F at the age of 14.5 years

Patient M (male) (Fig. 1); weight is 56.0 Kg (>97th percentile), height 168.0 cm (>97th percentile), HC 57.2 cm (98th percentile). Since the age of 2, this child has been in the 97th percentile for height. Parental heights: father 170.0 cm (20th percentile) mother 165.0 cm (75th percentile).

**Fig. 2:**
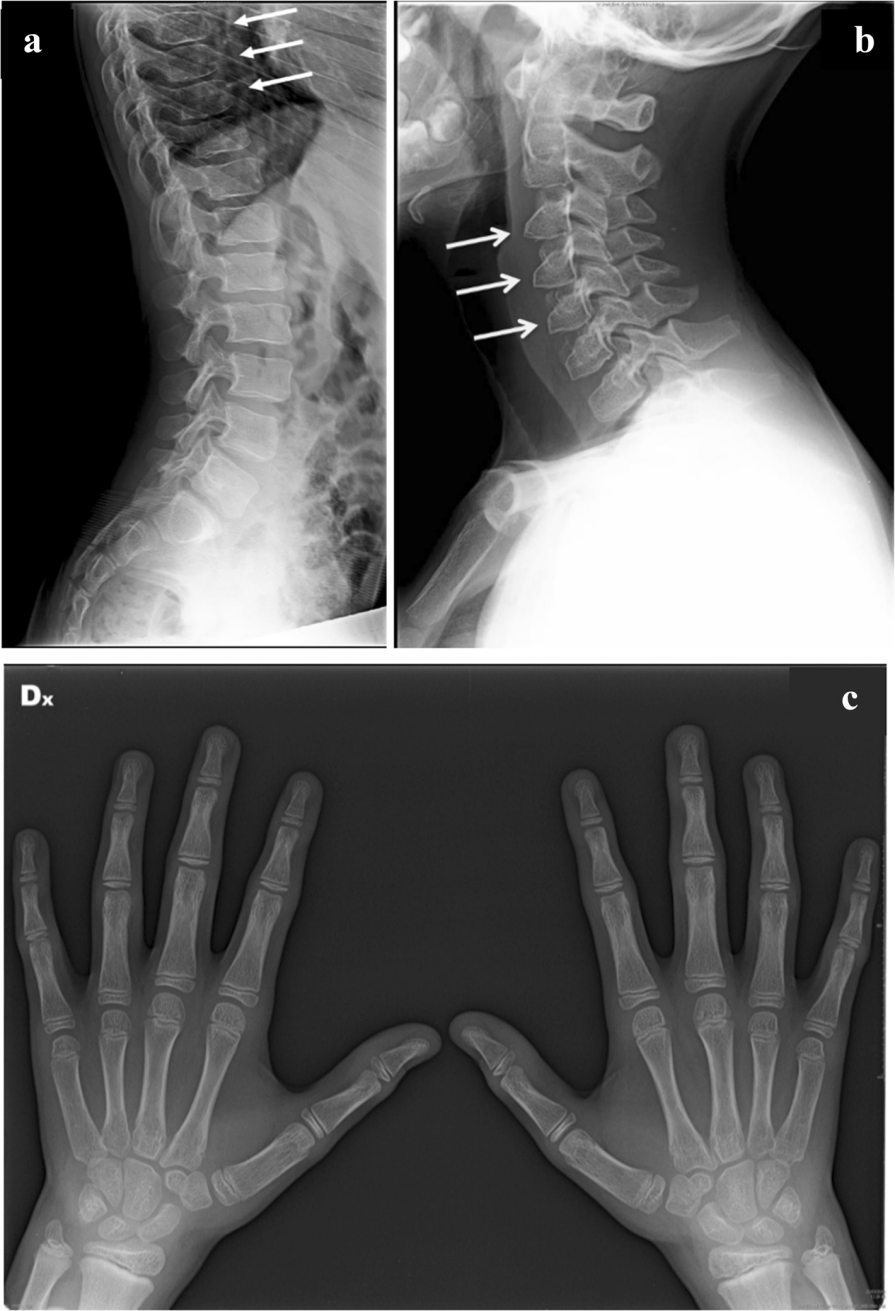
Patient M at the age of 11 years: (**a**) mild ovoid shape of low vertebral bodies; (**b**) mild triangular shape of cervical bodies; (**c**) no signs of multiplex dysostosis of the hands

At the age of 12 years this boy has not developed characteristic facial features of MPS I. He is intellectually normal (IQ: 116) with a quality of life (assessed by EQ-5D-Y) [4] of 100/100; no respiratory or sleep disturbances are reported; no hepato-splenomegaly at clinical examination. Musculoskeletal evaluation reveals evidence of mild limitation of flexion-extension of wrist (60/30; n.v. 75/70), ankle and of the 4th and 5th fingers bilaterally; shoulder and hip range of motion are normal and there is no evidence of scoliosis. Formal hearing assessment is normal, ophthalmologic evaluations have shown mild corneal clouding, first noted at 12 months of the age that has remained unchanged. From the age of 7 years he has developed mild hyperopia and astigmatism that is completely corrected by lenses. Echocardiographic evaluations demonstrated evidence of mitral and tricuspid valve thickening with mild insufficiencies, first detected at the age of 7 years; mild atrial dilatation was detected by age 9, but remains substantially unmodified to date (NYHA classification: I). Skeletal X-rays at age 10 showed evidence of minimal dysostosis multiplex of lower thoracic vertebral bodies (Fig. 2a) and triangular shape of some cervical bodies (Fig. 2b); hand X-rays were normal (Fig. 2c) and substantially unchanged. Bone densitometry is normal. Electroneurography shows bilaterally mild signs of median nerve suffering. Brain MRI is normal. No recurrent infections are reported. ABR and audiometry are within normal values.

Total urinary glycosaminoglycan (GAG) excretion were elevated before ERT and normalized after 4 months of therapy [3]. GAG determination on samples taken just before infusion remained within age related normal ranges until 8.5 years of age; at this time the levels increased to 89 μg/cr (nv: 37 μg/cr ± 18) by the age of 10.5 years, and subsequently normalized. Urinary GAG elecrophoresis, characterized before ERT by the presence of dermatan sulphate (DS) and heparan sulphate (HS), normalized after 4 months of ERT up to the age of 6 years, when the presence of DS and chondroitin sulphate (CS) with a ratio of 50/50 was observed up to date.

Plasma GAG determination at the age of 11.5 years was 2.8 μg/ml (nv: 2.7 μg/ml ± 1.12), with a CS/DS ratio of 98/2 (nv: 100/0); these are similar to levels obtained at the age of 6, 9 and 10 years. No pre ERT plasma sample was available. After 12 years of ERT circulating anti-laronidase antibodies were not detectable by ELISA methodology.

### Sibling 2

**Fig. 3:**
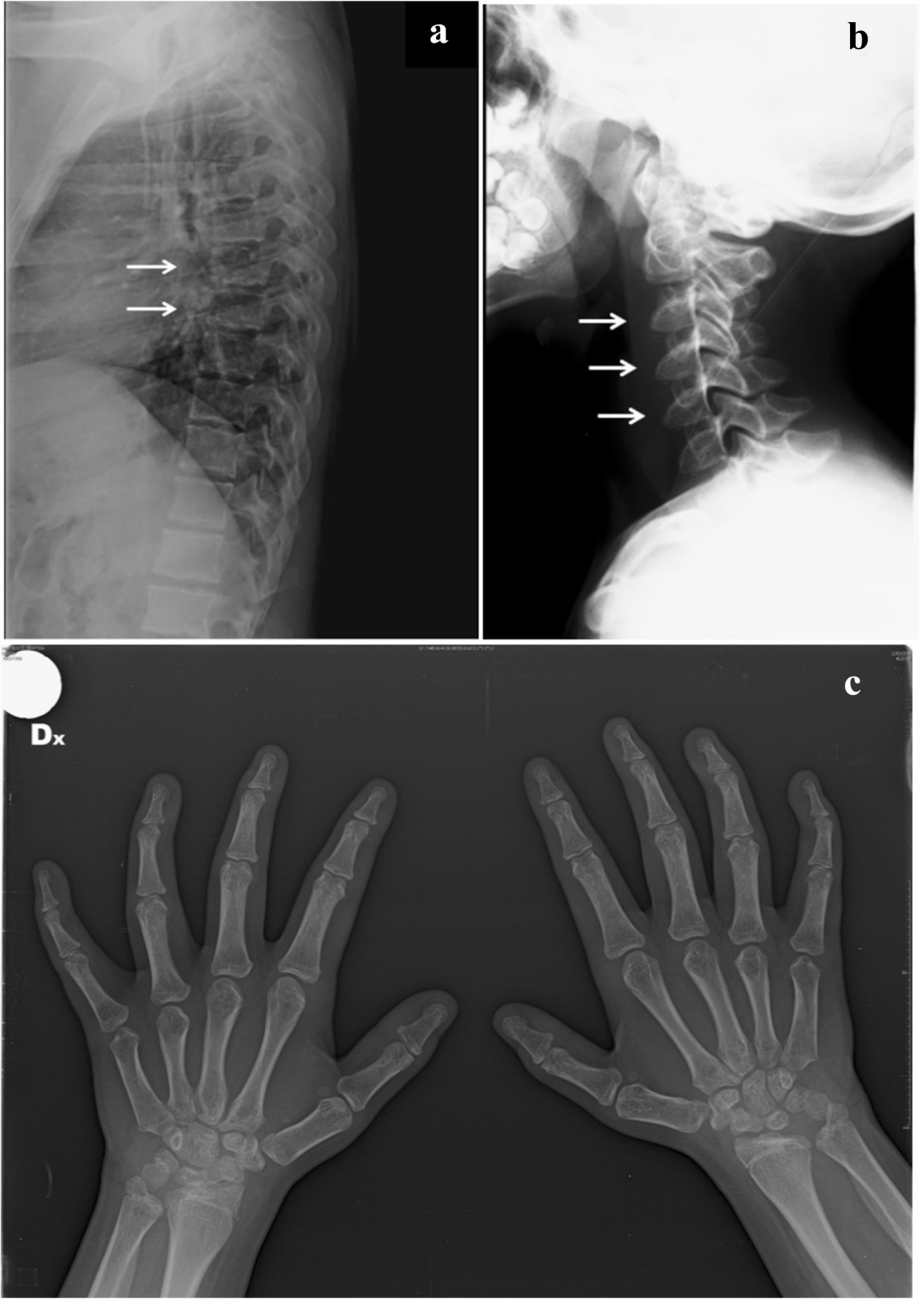
Patient F at the age of 16 years: multiplex dysostosis signs at thoracic (**a**) and cervical (**b**) bodies and hands (**c**)

Patient F (female) (Fig. 1) weight is 58.0 kg (>50th percentile), height 154.0 cm (10th percentile), and HC 59 cm (>97th percentile). She is intellectually normal (IQ: 80), with a quality of life (EQ-5D-Y) of 100/100; menarche began at the age of 12 years with a normal pubertal growth spurt. After 12 years of ERT she still has moderate facial coarseness (Fig. 1). Liver and spleen volumes normalized within the first year of ERT and have remained within normal limits at clinical examination. Musculoskeletal evaluation shows progression of joint disease with limitation of range of motion of both proximal and distal joints. She has developed contractures of all of her digits (claw hand deformity) and severe restriction of range of motion of all joints; in particular elbows, knees and ankles showed fixed flection to 15, 20 and 15°, respectively; the spine is straight. She cannot completely raise her arms above the head. Ophthalmologic evaluation reveals diffuse and prominent corneal clouding present since the age of diagnosis; the degree of clouding has remained unchanged after 12 years of therapy. At the age of 11 years she developed hyperopia and astigmatism, completely corrected by lenses (visual acuity: 10/10). She has had persistent recurrent otitis media and has mild bilateral conductive hearing loss persistent at the age of 16 years, confirmed by ABR and audiometry. No respiratory or sleep disturbances are reported. Cardiac evaluations demonstrated no significant progression of previously reported moderate mitral insufficiency with thickened valve leaflets, anterior edge prolapse and mild left atrium thickening (NYHA classification: II). Skeletal X-rays show no improvement of her moderately severe dysostosis multiplex with evidence of slight worsening of thoracic vertebral changes but stable cervical and hand findings (Fig. 3a, b and c). Signs of multiplex dysostosis were already present in the girl at the age of 4 years and 6 months [3]. Bone densitometry is normal for age. Spirometry yielded a mixed ventilator pattern (FVC 66 %, FEV_1_ 70 %, PEF 71 %, FEF_25–75_ 79 %); inspection of the flow-volume curve revealed abnormal flattening of the expiratory loop, which was suggestive of variable airway obstruction. Cerebral MRI shows no white matter alterations; electroneurography shows bilaterally mild signs of median nerve compression. Due to a progressive walking difficulty at the age of 16 years, a bilateral elongation of the Achilles tendon was performed.

Total urinary GAG excretion was only slightly above the upper limit of normal for age during the last 6 years. Urinary GAG electrophoresis showed the presence of DS and CS with a ratio of about 50/50 already observed at the age of 11 years [5]. HS, present before ERT, disappeared after 5 months.

Plasma GAGs at the age of 5 years, before ERT, were 18.2 μg/ml (nv: 5.27 μg/ml ± 3.01) with a CS/DS ratio of 25/75 (nv: 100/0); after 6, 9 and 10 year of ERT, plasma GAGs have remained within the normal range for age, with only traces of DS (CS/DS ratio of 98/2). After 12 years of ERT circulating anti-laronidase antibodies were not detectable.

**Fig. 4:**
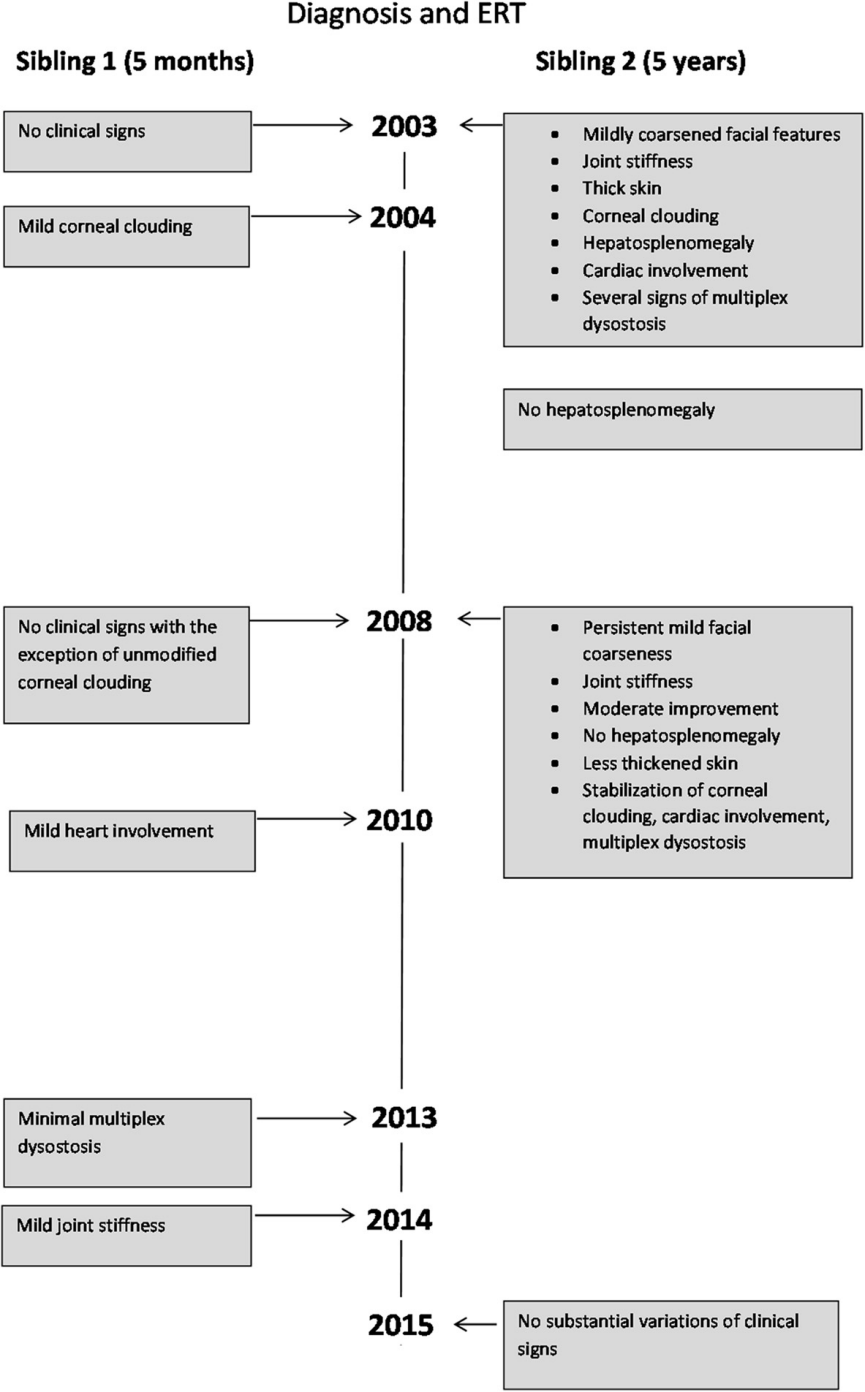
Timeline of follow up and the outcomes of the patients

The timeline of follow up and outcomes is reported in Fig. 4.

## Conclusions

Hundreds of MPS I patients have received ERT since the approval of laronidase [6–8]; however, the vast majority of them commence treatment when they have already a considerable disease burden. Published long-term follow up data show that although ERT is able to substantially modify the natural history of disease, it does have limitations in its ability to reverse key clinical manifestations [9–12]. Recently, these results have been also confirmed by Al-Sannaa N.A. et al. [13] on 4 patients treated during the first year of life. In fact, ERT is able to reverse some clinical signs such as thickness of the skin and hepato-splenomegaly, but ERT has limitation in relation to reversal of existing joint range of motion abnormalities. In addition, ERT is less efficient in altering other manifestations, once they are established, such as dysostosis multiplex, cardiac valvulopathology, and corneal clouding [7, 9], which result from a complex pathogenic cascades leading to irreversible damage of the connective tissue [11]. It appears that initiation of ERT when significant arthropathy is present leads to some improvement of joint range of motion in the first 1–2 years of treatment, followed by disease stabilization or a slower rate of disease progression. These observations have led to the assumption that very early initiation of ERT i.e. prior to significant manifestation of skeletal disease, may lead to improved long-term outcomes for patients. Similar arguments have been made in relation to the potential impact of early initiation of ERT on other key clinical signs such as cardiac, ophthalmologic and respiratory manifestations. The comparative observations after 12 years of ERT in this unique sibling pair provide convincing evidence of substantial differences in the impact of ERT in MPS I when therapy is initiated prior to significant disease manifestations. Notably, the skeletal, joint and cardiac valve involvement has continued to progress over eleven years of therapy in the sibling who started therapy at the age 5 years whilst the younger sibling, who started therapy at 5 months, shows minimal joint and cardiac disease with no evidence of progression of corneal disease. Despite the lack of symptoms related to skeletal involvement in the sibling who commenced therapy at a young age, he does indeed show evidence of thoracic vertebral involvement as well as cardiac valvular disease by the age of 7 years. Biochemical observations of this sibling pair reveal that later in childhood, a moderate increase of total urinary GAGs and more notably dermatan sulphate was observed. Moreover, normal level of plasmatic total GAGs were present, with only traces of DS. No HS, present before ERT, was detected both in urine and plasma. Recently, De Ru et al. [14] and Langereis et al. [15] reported that the presence of DS and HS could be related to the dose of ERT or to the antibody status of the patients. On the basis of these data and the clinical and biochemical results in our patients, we hypothesized that the amount of enzyme supplied could be one of the causes of the insufficient GAG degradation. Therefore, the appearance of clinical signs even after early therapy would indicate that either increased ERT dosing or additional adjuvant therapeutics should be considered. In fact, studies in MPS animal models indicate that early alterations of the extracellular matrix and inflammatory mechanisms likely play a role in the progressive skeletal and connective tissue disease [16, 17]. Therefore, the addition of adjuvant therapies targeted to these pathways or therapies targeting substrate reduction may be required in order to afford the best outcome for patients.

This case report illustrates that early treatment substantially modifies the natural history of the attenuated form of MPS I, and brings to the forefront the importance and potential impact that neonatal screening and early treatment initiation could have for this progressive disease.

## Consent

The family was informed of the nature of the study, and written informed consent was obtained from the parents and the 17 year old daughter for publication of this case report and any accompanying images. A copy of the written consent is available for review by the Editor of this journal.

## Abbreviations

ERT: Enzyme replacement therapy
MPS I: Mucopolysaccharidosis type I
GAG: Glycosaminoglycan
DS: Dermatan sulphate
CS: Chondroitin sulphate
